# Functions of Music Making Under Lockdown: A Trans-Historical Perspective Across Two Pandemics

**DOI:** 10.3389/fpsyg.2020.616499

**Published:** 2020-12-17

**Authors:** Remi Chiu

**Affiliations:** Department of Fine Arts, Loyola University Maryland, Baltimore, MD, United States

**Keywords:** plague, COVID-19, emotions, ritual, social cohesion, music, quarantine

## Abstract

This paper describes how music fulfills two of its broadly recognized functions—“mood regulation” and “social cohesion”—in times of pandemics and social isolation. Through a trans-historical comparison of the musical activities of the Milanese during an outbreak of plague in 1576 with the musical activities observed during the COVID lockdowns in 2020 (such as balcony-singing and playlist-making), this paper suggests a framework for understanding the role of music in the care of the biological body and the social body in times of medical disaster.

## Introduction

Writing for *Critical Inquiry*’s blog at the beginning of the near-global COVID lockdown in April 2020, Lorraine Daston observed that we have been “temporarily thrown back into a state of ground-zero empiricism,” a state of great scientific and epistemological uncertainty where “chance observations, apparent correlations, and anecdotes that would ordinarily barely merit mention, much less publication in peer-reviewed journals, have the internet buzzing with speculations among physicians, virologists, epidemiologists, microbiologists, and the interested lay public” (Daston, 2020). Professional historians, journalists, and other humanists interested in the social responses to epidemics, too, have been buzzing. In the shock of novelty and uncertainty, of having nothing quite like the current situation in our collective memories, we have exhumed narratives of past medical calamities—the 1918 Flu pandemic, for example, or the three pandemics of plague—in search of cultural signposts for the present. We have rediscovered that some Americans were also wearing their masks improperly during the flu pandemic (Burch, 2020); Daniel Defoe also lamented that the poor appeared far more vulnerable to plague (Jordison, 2020); Samuel Pepys wore his new periwig only after quarantining it for a good while, as some of us had done with our deliveries (Bowyer, 2020); and Boccaccio told us that the medieval well-to-do, like ours, happily fled to their country homes (McKinley, 2020).

We can also find in past epidemics some antecedents and analogs for the musical activities, such as therapeutic music making and balcony flash mobs, that emerged during the COVID-19 lockdowns. This paper will examine, from a musicological perspective, different kinds of musical activities across two pandemics—the second pandemic of plague and COVID-19—and what they can tell us about the functions of music in times of extraordinary danger and isolation, as well as the practices, technologies, and medical and cultural beliefs that enable music to fulfill those functions.

There are good reasons, certainly, to be skeptical of facile trans-historical analogies. In *The Lancet*, medical historian Robert Peckham, writing of the COVID-19, warned that “when the present is viewed through the lens of former disease outbreaks, we typically focus on similitudes and overlook important differences” (Peckham, 2020, p. 850). Eisenberg et al. (2020) cautioned that treating the coronavirus like previous plague epidemics may be dangerous in part because many aspects of past epidemics still remain mysterious. And in the studies of the artistic responses to past epidemics, grand narratives of personal creativity and psychological influence are often built on shaky foundations. Art historian Millard Meiss, for example, perhaps influenced by his own experiences of the Great Depression and WWII, offered the once-influential theory that the trauma of the Black Death produced a mood of penitence and mystical fervor among Florentine and Sienese artists, who turned away in pessimism from a progressive naturalism, human expression, and realistic narrative, toward a backward-looking style of hieratic and abstract forms associated with the thirteenth century (Meiss, 1951/1973). In later research, Cohn (1996) countered that the stylistic conservatism Meiss noticed (if they truly existed at all) could have been necessitated by a surge of demand for memorial art in central Italy—including funerary monuments, chapels, paintings, engravings, and decorative arts—evidenced by the extant wills and testaments that commission them. The “boom” in demand, first after 1348 and especially after a second strike of plague in 1363, came surprisingly with a corresponding drop in the price of commissions to the lowest level in the fourteenth century, even as costs in general rose amid economic disruptions. This picture of cost and demand suggests that patrons, perhaps unable to pay higher prices, were interested in simpler images, produced quickly and cheaply. Moreover, as Steinhoff (2007, p. 3–26) has noted, newly formed collaborations between ateliers in response to uncertain market conditions necessarily disrupted the earlier norms of stylistic unity. Economic factors, then, rather than psychological change—which is difficult to divine for individual historical actors and often just as difficult to discern for victims of trauma—may have been the root cause of perceived epochal style changes. These are, of course, the same methodological difficulties in historiography *tout court*, but the rush of information in this COVID ground zero—the chance observations and anecdotes—can increase the challenge.

On this difficult terrain, I will focus on musical events and their contexts in the 1576–1578 plague outbreak in Milan that can help frame our consideration of the musical practices and their functions under COVID today. That outbreak in Milan, also called the Borromean Plague or the Plague of San Carlo in honor of the Archbishop Carlo Borromeo, who shepherded the city through the crisis, is useful because it was among the first to be described and chronicled in some detail by non-medical professionals (Cohn, 2011, p. 6). The documents these writers left behind provided valuable information about public-health and sociocultural reactions against the outbreak that was not easily available in earlier, more medically oriented writings about the plague. The medical and social responses to plague in turn impacted the practices of music making in such extraordinary times.

Looking across pandemics—and reconciling necessarily different kinds of documentary evidence, from chronicles and medical treatises in the past to news reports, interviews, and empirical experiments in the present—there are two functions of music that appear particularly valuable in times of medical disaster and isolation: “mood or affect regulation” and “social cohesion.” These are functions of music, in general contexts, that have been broadly recognized by psychologists today (DeNora, 2000). In their literature review and survey of listeners, for example, Schäfer et al. (2013) identified those two categories, along with “achieving self-awareness,” as the main functions of music. Younger music listeners have been shown to value those two functions of music in particular (Groarke and Hogan, 2016). Thayer et al. (1994) found “listening to music” to be the third most frequent tactic employed by survey respondents to improve a bad mood, after talking or being with someone and controlling their own thoughts, and second most successful for achieving that goal, after exercise. Similarly, van Goethem and Sloboda (2011) found that music was, after talking to friends, the second most used affect-regulation tactic in everyday life and nearly as successful, and the most frequently used tactic for the goal of creating calm/relaxed and happy/excited affects. Saarikallio (2011) found that subjects tended to choose music based on their current moods for the goal of mood improvement, but when they selected music consciously and specifically to regulate their moods, the target mood typically related to physiological arousal (e.g., to relax or perk up). Finally, a variety of strategies for musical mood regulation have been proposed, including relaxation, distraction, active coping, introspection, venting, and rational thinking (van Goethem and Sloboda, 2011; Sakka and Juslin, 2018).

Additionally, “affect regulation,” under different epistemologies about disease, emotions, and psychosomatic phenomena, remains tied to physical health across historical periods. The fifteenth century Neoplatonist philosopher and doctor Marsilio Ficino, for example, advocated singing melodies sympathetic to a person’s temperament in order to prolong life (Ficino, 1989). Such a prescription was predicated upon a belief that there was a network of interconnectedness among all things. That music can affect the human body and soul betokened those hidden connections and operated on the same principle as sympathetic vibration, a demonstrable phenomenon whereby if one of two similarly tuned strings in proximity to each other is plucked, the other would vibrate spontaneously. Today, biochemistry has replaced resonance and hidden sympathies in the explanations of music’s relationship to mind-body interactions. Fancourt et al. (2014), for example, have surveyed the increasing amount of literature describing the psychoneuroimmunological responses that arise from listening to and performing music, though in many cases, the exact mechanisms by which music achieves neurological and immunological effects, as well as the relationships between the biomarkers affected by music and the body’s immune functions still remain unclear. Research on music as analgesic likewise establish the somatic consequence of music’s ability to regulate affect, with some work suggesting that music can relieve pain by interrupting the pain-stress feedback loop (Bernatzky et al., 2012; Linnemann et al., 2015; Basiński et al., 2018).

Music has been found to foster social cohesion in numerous ways by researchers who invoke a variety of theoretical mechanisms to explain this musical function, from mirror neurons, to hormones, to semiotics (Tarr et al., 2014; Clarke et al., 2015). Hove and Risen (2009), for example, found that participants who were given the task of tapping in synchrony with an experimenter reported a greater sense of affiliation with her than those who did not, concluding that “in a world rife with isolation, the aligned representations in interpersonal synchrony may provide a means for togetherness and connection.” Keller et al. (2014) have argued that the cognitive-motor processes and neurophysiological mechanisms required in joint rhythmic action (such as marching, military training, religious rituals, dance, or music) lead to “greater social cohesion, cooperation and trust” among participants. Group singing, especially improvisatory singing, has been found to encourage the production of oxytocin, which can promote interpersonal trust and attachment (Keeler et al., 2015). In a study of the symbolic value of music, Boer et al. (2011) have noted that similarity in musical taste can function as proxies for perceived similarity in value orientations that contribute to social attraction between subjects.

Evolutionary hypotheses of music’s adaptive function also focus on both its capacity to moderate emotion and to support social bonding, often with those two functions working in tandem, since music making in hunter-gatherer societies tended to occur in social contexts. For example, lullabies, found in many cultures, reduced psychological distance between parent and child, and may have enhanced infant survival in ancestral conditions by pacifying them (Trehub, 2001). In adulthood, music making may have arisen as courtship behavior that advertises good health, and in larger groups, music could promote pro-social behavior by regulating moods and actions. For example, singing and dancing prior to a battle may raise the arousal of warriors and synchronize their individual moods to serve the larger goal of the group (Huron, 2001; Schäfer et al., 2013). If such speculations are correct, then the mood regulation and social cohesion functions of music are deep-seated and would be particularly relevant in times of increased emotional turmoil and social isolation, such as during epidemic outbreaks.

## *Yersinia Pestis*, the Plague, and SARS-CoV-2

Historians have grouped plague epidemics into three broad pandemics: the first, 541-750 CE; the second, 1346-ca. 1850; and the third, 1855–1950. There is a consensus among historians and forensic archeologists that the disease historically called “the plague” is caused by the bacterium *Yersinia pestis*, discovered during the third pandemic (Bolton, 2013). The most common and characteristic form of plague is bubonic, so-called on account of the buboes that form in the lymph nodes. Bubonic plague is caused by the bite of an infected rodent flea. If the disease enters and accumulates in the bloodstream, then septicemic infection can occur. This is a rarer form than the bubonic, but far more dangerous; left untreated, death is certain. The third form of plague, affecting the lungs, is pneumonic plague. This form of plague is as deadly as septicemic plague and is the only form that can spread between humans via infectious droplets. However, evidence suggests that pneumonic plague is not easily transmitted, historically with an *R*_0_ value near 1 (Kool and Weinstein, 2005). For comparison, in June 2020, the WHO estimated the *R*_0_ of SARS-CoV-2 to be between 2 and 4.

However, certain we may be that *Yersinia pestis* was the same causative agent of all three of the historical plague pandemics, the experience of plague was certainly very different before the development of modern germ theory. For the Milanese assailed by plague in 1576, the disease was not caused by bacteria, but by putrefied air (or “miasma”) that is absorbed through the lungs or the pores, which then corrupted the blood, one of the four humors—along with phlegm, yellow and black biles—that must be maintained in balance with the others to ensure good health. Miasma, in turn, could be caused locally by improperly disposed fetid materials or at a more “universal” level by events that changed the quality of the air over vast regions, such as the inauspicious alignment of heavenly bodies or vapor-releasing earthquakes. Ultimately, and especially in the case of these latter events that generated large-scale epidemics, plague was the work of a vengeful god, angered by the sins of his people and enacting his punishment through nature. One fifteenth century doctor succinctly summarized this etiology of plague: “God is the most remote cause of epidemic disease, the heavens are the more remote, the air is remote, the humor is near, putrid air is nearer, and the putrid vapor infused in the heart is the nearest” (Johannes de Saxonia, 1924).

While miasma was theoretically an “environmental” problem, a large majority of doctors treated it as a contagion that can be passed through insalubrious exhalations of the sick, trapped and transported in goods and materials, and even spread via touch. While doctors at the time lacked a coherent theory of contagion, it was observed from the start of the second pandemic that plague could be transmitted from person to person. A Pisan chronicler recorded that during the 1348 outbreak, “when [the crew of two Genoese galleys] reached the fish market someone began to talk with them and immediately he fell ill and died; others who talked with them also became ill as well as any who were touched by those who had died… and thus was sparked the great corruption that killed everyone” (Cohn, 2013, p. 112). The drastic custom of boarding up the sick along with their otherwise healthy cohabitants inside their homes (Newman, 2012) speaks to a fear of contagion that appears incommensurate to a disease that, as observed during the third pandemic at least, does not readily spread between people.

## The 1576 Plague of Milan

It is the miasma-contagion model of plague, rather than the clinical knowledge of *Yersinia pestis*, that precipitated the same kinds of civic health responses during the second pandemic that we see today with the coronavirus. Because premodern plague and the coronavirus are both primarily “aerial” and contagious, a large proportion of public health measures against both of these diseases focused on the sanitation of the air, isolation of the sick, and the control of contagion. The Milanese response to the Borromean Plague outbreak is illustrative in that regard.

Plague was first discovered within Milan’s walls in late-July, early-August 1576. By that point, the surrounding regions of Trent, Mantua, and Venice were already stricken. How the disease entered the city was a topic of much rumor and speculation. Some thought the culprit was a woman from Marignano, others thought it was a nobleman from Mantua, while others still pointed to villains who were seen smearing pestiferous unguents onto city gates. The Milanese governor and the nobles abandoned the city as disease spread through every neighborhood. By September, as many as 300 citizens were dying daily, and the death toll increased into mid-December. The sick as well as those suspected of being sick were sent to the city’s lazaretto (plague hospital), and their homes were purged and cleaned. The lazaretto, a large compound of 288 rooms surrounding a large central campground where a church stood, was quickly inundated. Over 3,000 temporary huts had to be built outside the city gates; hundreds more were needed, but the city ran out of straw. Beginning on 29 October, Milan was placed under a general quarantine. Dispensations were granted to certain essential shops to open, and heads of households had to obtain written licenses to leave their homes (Bisciola, 1630).

According to the accounts, Carlo Borromeo not only provided pastoral guidance, visiting the sick and holding citywide devotional rituals, but he took charge of civic matters as well. The Archbishop spent his own funds to improve the lazaretto and, along with the city’s council, contributed to the city’s provisions when industry and trade quickly dropped off (the Spanish government was unwilling to send monetary aid to the city because of its ongoing and costly military commitments elsewhere). After a slight decrease in the number of deaths, plague resurged in February 1577, and it was not until the end of March that quarantine measures were slightly relaxed to allow men and women to go to church over the Easter season. By May, the citizens were cautiously optimistic that the tide had turned and began to venture onto the streets again (Bisciola, 1630). Nevertheless, the Milanese were cautious and would not declare their city plague-free until January of 1578. The final death toll according to the Milanese health board was 17,329, or around 14% of the city’s population (D’Amico, 2000, p. 4).

## Music at the Lazaretto

The etiology of plague and the resultant public health responses all had an impact on music making in times of plague. An episode at the Milanese lazaretto brings up some of the ethical problems of music. At the start of the outbreak, the Capuchin monk Fra Paolo Bellintano volunteered to be the warden of the hospital. Describing the hospital as a “den of thieves,” he recounted in a dialogue an episode concerning a fellow Capuchin, brother Andrea of Bione, who shared the burden of ensuring the moral hygiene of their charges:

One night the inmates were staging a dance, in order to cheer themselves up, keeping the event secret even though I forbid all such activities. The day before, brother Andrea had recounted to me that he had seen among the cart of dead bodies a very old woman, and knowing about these festivities, he planned to instill a bit of terror among the dancers. He went that night to the pit in the middle of the lazaretto where they threw corpses, and searching among them diligently, finally found her again…He said, “Quiet old girl, we’re going to a dance.” And he went into the room, knocked on the door, and announced, not as friars do with “God bless,” but, “Let us in, we’ve come to party!”

When they opened the door, he hurled the body into the middle of the room saying, “Let her dance, too.” Then he added, “Is it really possible you will stay here debauching, offending God, when your deaths are so close at hand?”… The dance ended (cited in and adapted from Carmichael, 1998, p. 153).

Whether this party actually took place or not, Fra Paolo’s anecdote nevertheless speaks to a broader ambivalence in some sectors toward musical recreation during epidemics. For those who were religiously inclined, music *per se* as well as its associated revelry can lead to sin and exacerbate God’s ire. Such suspicions toward music were deep-rooted; as early as the fourth century, Augustine worried that music—even religious songs—appealed too much to the senses and the flesh.

Musical recreation was especially problematic in a religious (or religiously operated) institution such as the lazaretto, where the duty of care applied to both body and soul. The very panoptic layout of the Milanese lazaretto, with the rooms built around a church in the center of the campus, promoted spiritual discipline. Carlo Borromeo and the Capuchins even viewed the plague hospital as a kind of moral testing ground: Borromeo exhorted those in the lazaretto and under curfew to “prepare themselves to use this time well, and treat every day of this quarantine as being like the holy time of Lent: and just as Our Lord fasted for forty days in the desert” (cited in Crawshaw, 2012, p. 47). Borromeo’s invocation of Jesus’s time in the desert was particularly apt in this context, as “quarantine” referred to a period of isolation of forty days, a length of time rife with religious significance (Lent, the Great Flood, Moses’s stay on Mt. Sinai; Sehdev, 2002).

Spiritual concerns notwithstanding, staging a dance “in order to cheer themselves up” was precisely what doctors of the time would have ordered. Many Renaissance doctors, like Niccolo Massa, warned against negative affects in their plague treatises and prescribed music and other gentle recreations as an anodyne:

Many people, from fear and imagination alone, have fallen to pestilential fever; therefore, it is necessary to be joyful… One should stay in a bright and well-decorated home…with scents and fumigations. Or take a walk in a well-appointed garden, since the soul is restored by this. Furthermore, the soul gladdens in meeting dear friends and in talking of joyful and funny things. It is especially advantageous to listen to songs and lovely instrumental music, and to play now and then, and to sing with a quiet voice, to read books and pleasant stories, to listen to stories that provoke moderate laughter, to look at pictures that please the eyes… (Massa, 1540, 39r).

Such prescriptions that placed music alongside food, drink, rest, exercise, and sleep in the anti-pestilential regimen were numerous across the fifteenth and sixteenth centuries, and they reflected the belief in a strong psychosomatic bond that held together the mind and body. It was thought that the imagination (*imaginatio*)—a mental faculty that hosted sensations and memories—can call up powerful emotions that can perturb the body (Harvey, 1975, p. 44–6). Gaspar Torrella, for example, describing the oft-experienced effects of chills and mental confusion on account of worry and fear in premodern medical terms, writes that those emotions move the body’s vital heat and spirit inward rapidly, freezing the entire body and dimming the mind (Torrella, 1504, B2v). The imagination being so powerful, it was commonly thought that the mere thought of plague was enough to bring on buboes on the body. Musical recreation, under this scheme, can occupy the mind and effect the salubrious joy that counteracts such noxious imaginings (Chiu, 2017, p. 12–9).

But lest we should think that the ethical ambiguity concerning music came about as a simple opposition between secular medical practitioners and ascetic religious authorities, the doctors themselves subscribed to the etiology of disease whereby God was the ultimate cause. There were a few religiously inclined doctors, such as Giovanni Filippo Ingrassia, who questioned the value of music. Writing during an outbreak in Palermo that occurred at the same time as the plague of Milan, he confessed that he did not wish to follow the advice to “attend banquets, enjoy pleasurable pastimes with friends, games, witty conceits, laughter, comedy, songs, music, and other such nonsense.” With so many dying in the space of a few days without confession or other sacraments, he asked rhetorically, “What blind mole could, in such a situation, be happy and carefree, mindlessly living like Sardanapalus?” (Ingrassia, 2005, p. 441).

Taken together, Niccolo Massa and Ingrassia provided two ethical views of musical activities that Bocaccio had also described in the fourteenth century. In the *Decameron*, he distinguished between two kinds of Florentines who would indulge in music during the plague:

Some people were of the opinion that a sober and abstemious mode of living considerably reduces the risk of infection. They therefore formed themselves into groups and lived in isolation from everyone else… They refrained from speaking to outsiders, refused to receive news of the dead or the sick, and entertained themselves with music and whatever other amusements they were able to devise.

Others took the opposite view, and maintained that an infallible way of warding off this appalling evil was to drink heavily, enjoy life to the full, go around singing and merrymaking, gratify all of one’s cravings whenever the opportunity offered… They would visit one tavern after another… But for all their riotous manner of living, these people always took good care to avoid any contact with the sick. (Boccaccio, 2003, p. 7)

Without taking on an overtly religious tone, Boccaccio implicates music in a variety of behaviors—on the part of those who “bubbled” with loved ones and those who caroused publicly but were otherwise careful about avoiding the sick—that were either moderate and merited praise or else immoderate and deserved moral censure.

Today, with an increasing awareness that psychological stress can have deleterious health effects (Segerstrom and Miller, 2004; Morey et al., 2015; Mroczek et al., 2015; Picard and McEwen, 2018; Trudel-Fitzgerald et al., 2019), the regulation of mood through recreation and socializing has found its way into the anti-COVID regimen. Recall Niccolo Massa’s prescription to avoid negative affects such as fear and worry, take walks in gardens, talk with friends and loved ones, and engage in gentle recreations. The American national health agency Centers for Disease Control and Prevention has provided strikingly similar guidance on their website for “healthy ways to cope with stress” during the pandemic that includes taking breaks from “watching, reading, or listening to news stories” because “hearing about the pandemic repeatedly can be upsetting”; taking time “to unwind” and trying to engage in enjoyable pastimes; and connecting and talking “with people you trust about your concerns and how you are feeling” (Centers for Disease Control and Prevention, 2020).

But, as in the past, there is a divide between what is considered salubrious and desirable contexts, and frivolous and dangerous contexts for music. When 246 primary and secondary cases of COVID-19 in South Korea were traced to nightclubs that catered to an LGBTQ clientele (Kang et al., 2020), club-goers were broadly denounced and homophobic hate speech arose (Kim, 2020). College students’ activities were policed (with peer and community surveillance, and threats of fines and suspensions) when American post-secondary institutions reopened in the fall, and their behavior criticized by the public and by institutional leaders when outbreaks occurred. For example, Gordon Gee, the President of West Virginia University, which suspended in-person classes early September after a brief reopening, expressed disappointment in an official statement after seeing “the photos and commentary that are circulating on social media showing West Virginia University students gathering outside local bars” (Gee, 2020). In contrast, in response to reports of super-spreading events at multiple choral rehearsals and performances (see Hamner, 2020, for example), agencies of various kinds—including the Freiburg Hochschule für Musik, Emory Voice Center, National Federation of State High School Associations, College Band Directors National Association, and the American Choral Directors Association—quickly began to study the dangers of singing, with the goal of providing guidance on how to continue ensemble music making safely. While the force of modern criticism against revelers stems from our fear of contagion rather than moral objections against dancing and music *per se*, there remains, for us as for Boccaccio, differing levels of tolerance for different kinds of musical activities that are imbricated with wider and long-standing moral, economic, and political concerns.

## Music on the Streets

While Borromeo and other spiritual authorities were suspicious of recreational music, they nevertheless made use of religious music in the context of organized rituals and private devotion. Among the most common devotional responses to civic crises, including the plague, were citywide processions, the primary purpose of which was for a community to acknowledge their sins and demonstrate their contrition to God publicly and openly. Certainly, civic health boards and even spiritual authorities themselves understood how dangerous these processions were. Echoing contemporary conflicts between churches defending their right to assembly and public health officials wary of congregations, debates about safety among those groups in the Renaissance were sometimes contentious. There were a few instances where processions were outright banned, the numbers of participants limited, or their scope and spatial reach curtailed. During the 1576 outbreak in Venice, for example, processions were limited to the piazza San Marco (Cohn, 2011, p. 129). In Milan, when the situation appeared to be improving in May 1577 and curfew was gradually relaxed, citywide processions were held without the participation of the women, who were kept at home (Bisciola, 1630). Broadly, processions in some form were usually permitted because they brought comfort to the people and were deemed vital in addressing divine wrath, the ultimate cause of plague.

In early October 1576, Borromeo held three citywide processions in Milan that were typical of this kind of devotion across Catholic Europe. The different parishes gathered at the city’s cathedral and, each day, marched to a different terminus. As they processed, they sang a litany, a simple call and response prayer wherein one group would call out the name of a holy figure or a specific petition (“Saint Sebastian,” or “Free us from plague,” for example), and another group would respond “pray for us,” “intercede for us,” etc. The repetitive litany was simple enough—musically and linguistically—that Borromeo could send his followers out to teach the poor to sing the chant. Passing back and forth through the throngs of penitents, the litany would only be interrupted when the participants passed by a shrine or a church along the way and stopped to pay honor to their patrons.

Although the general course of these rituals was the same across Catholic Europe, their details were heavily customized to be meaningful to specific communities. Most fundamentally, the routes of the processions and the landmark stops were necessarily different from city to city, so the itineraries reflected the unique physical and cultural geography of a given place. The relics that were carried in procession were chosen for their importance to the community and articulated a shared civic history. While Borromeo brought out the nail of the holy cross in his 1576 processions, for example, it was his own exhumed body that was carried in procession during the 1630 plague outbreak in Milan, in honor of his good work during the previous crisis. Finally, as with the choice of saintly relics taken onto the streets, the litany itself was customized with added names of local intercessors. In the 1576 processional pamphlet published in Milan for use during that plague crisis, for example, minor figures such as Nazarius, Sisinnius, and Calimerus were inserted among more “universal” names like Peter because of their Milanese ties. On the whole, then, while processions were religious rituals in the first instance, they also functioned as civic rituals that fostered social solidarity through communal action and the projection of a shared devotional culture (Chiu, 2018).

While large-scale, city wide processions are no longer a mainstay of public health, religious anti-COVID processions still take place today in attenuated forms, with the same intentions for divine intervention and practices that evoke collective memory, and governed by the same kinds of concerns about contagion. In March 2020, for example, the Vysokopetrovsky monastery in Moscow, invoking the long history of religious crisis processions as established in Byzantium, announced daily processions to ward off the virus (Carroll, 2020). The clergymen walked around the perimeter of their monastery, carrying a copy of the Blachernae icon, singing, and sprinkling holy water on the monastery walls. In the announcement for the ritual, the abbot invoked the Lord, The Virgin Mary (Theotokos), and the local saint “Peter of Kiev, the Moscow Miracle Worker,” for aid. The number of participants were limited to eight clerics only, and lay parishioners were barred to mitigate contagion risk. In Rome, anti-COVID symbolism accrued to the Good Friday procession that recreates the Stations of the Cross, which had been relocated from the Colosseum to St. Peter’s Square (D’Emilio, 2020). A handful of participants, including a pair of Vatican doctors and some nurses representing the community of frontline health workers, processed around the otherwise empty square, while Pope Francis prayed before a crucifix that had been carried in procession during a sixteenth century outbreak of plague in Rome.

Religious institutions today have made use of the litany as well. The U.S.-based internet Catholic radio station Relevant Radio, for example, broadcast the “Litany in Time of Need” daily during the lockdowns in hopes of securing divine intervention. In this litany, led by Archbishop Bernard Hebda, the spirit of customization is still evident: a section of the roll-call of names included holy figures known for the care of the sick, such as the plague protector St. Sebastian, St. Borromeo, and Blessed James Miller, who had ties to Minnesota, where Hebda ministers. Importantly, in the introductory preamble to the broadcast, the litany is described as a “communal” prayer (Hebda, 2020). Litanies from other Christian traditions in the United States that do not focus on saints likewise serve a communal function by directing prayers to specific groups such as “the furloughed workers,” “the technologically isolated,” and “doctors, nurses and all health-care workers” (Burdette, 2020; Campbell, 2020).

## The Pleasures of Lists

The mood-regulating benefits of singing and the social cohesion (as well as the potential thaumaturgic power) of the litany aside, the enumeration of protectors in the prayer might have served another kind of mood-enhancing function. Semiotician Umberto Eco has argued that act of listing and collecting things that are potentially innumerable can inspire a psychological pleasure. Taking as an example a silver statue of St. Charles Borromeo in the Milan Treasury, which has been decorated with an accretion of gems from various patrons and donors, Eco suggested that the overall effect of sumptuous wealth built up from this accretion must have brought a sort enjoyment to the original collectors “where the pleasure taken in the precious material is indistinguishable from the aesthetic pleasure taken in the form given it” through collecting (Eco, 2009, p. 173–77). Likewise, for Christians performing litanies, Eco argued, the details of which holy figures were included or excluded may have mattered less than the sheer sustained enumeration of them (Eco, 2009, p. 118).

Today, we can find an expression of such pleasure of collecting in playlist making, a kind of musical activity that provided mood regulation and social cohesion during COVID lockdowns. A part of the enjoyment of creating playlists, McCourt (2005) has argued, comes from the symbolic sense of ownership that one gets when customizing and trading these lists, as music becomes less and less tied to material objects such as CDs and LPs. The curated music itself can provide other pleasures. In his ethnographic work on the iPod, Bull (2005) identified a number of functions that playlists serve for their creators, chief among which are “mood maintenance” and “recall of memories.” Anja Hagen’s observation of students’ use of the playlist function on the streaming service Last.fm ties together these two functions under the heading of “control”; she concluded that playlists “curated by moods, feelings, memories, or biographical/relational representations help the user experience mastery over the self” (Hagen, 2015, p. 642).

On popular music-streaming platforms such as Spotify, there is an innumerable amount of COVID- and quarantine-related playlists created and shared by users (an impression of abundance reinforced by Spotify’s “infinite” scrolling web interface). Curators of COVID playlists reported precisely those functions—control, recall of memories, and mood maintenance—when explaining their inclusion of particular songs. One of the contributors to the “COVID-19 MixTape” hosted on Spotify selected “Spaceship” by Galantis because the song “brings back memories of a good time with people at concerts and lounges.” Another contributor to the same playlist chose “(Don’t Fear) the Reaper” because it “takes me back to high school—it fought off the fears of the last years of the cold war as we faced our future. It felt like my friends and I were invincible!” (Hung, 2020). Others have published playlists curated by moods such as joy—which frequently include up-tempo songs, songs with encouraging lyrics, dance music, and even spiritual songs—and even melancholy (see Sachs et al., 2015 for a literature review on the pleasures of “sad” music). In a media release from 30 March 2020 (“How Social Distancing Has Shifted Spotify Streaming”), Spotify reported that users were adding more “chill” music to their playlists (not necessarily COVID-themed ones) than they had previously—more acoustic, more frequently instrumental, less danceable, and lower energy. COVID-themed Playlists that provide “gallows humor” are also common—songs such as Billy Idols’ “Dancing with Myself” or Britney Spear’s “Toxic” frequently appear. Along that vein, Spotify reports that The Police’s “Don’t Stand So Close to Me” had a 135% spike in streams in March. And while salons remained closed, Spotify reported on May 27 (“The Most-Streamed Songs and Podcasts of Summer 2020”) that there was a 50% increase between April 17 and May 17 in the creation of playlists using hair-related keywords. Performed by a Beneventan balcony flash mob (see below), the tamurriata “Vesuvio,” with such lyrics as “Is this a place for homes or jail where you are shut in from morning until night?,” provides the same sort of humorous irony. “Practical” lists (what Hagen calls context-sensitive playlists) of music to accompany activities such as painting, baking, and working from home have also increased by as much as 1,400% (Spotify, 27 May 2020).

But what made playlists particularly valuable during COVID isolation was their social functions. As more and more countries entered lockdown, Spotify reported on March 30 an increase in collaborative playlist making, “allowing people to connect over shared music and have virtual jam sessions together.” And as McCourt (2005) explained, aesthetic communities or “taste tribes” can form around online playlists (potentially indicating social attraction among members; Boer et al., 2011), so that single-curator lists reflecting personal tastes are also social objects if they are created with an imagined audience in mind or shared publicly. In the same media release, Spotify also noted that their users were sharing “more content on their social networks than usual, so they can let their friends and followers know what they’re up to from afar.” Just as the Milanese created a sense of shared culture with fellow citizens through their musical enumeration of saints, playlist makers today cultivate a community, unbound by geography, through shared musical choices.

## Music on the Balconies

Another group of songs that have seen spikes in streaming figures are ones that have been used in balcony performances, the recordings of which have circulated widely on social media. According to the March 20 report by Spotify, streams of two of the songs sung by Italian flash mobs—“Abbracciame” and “Azzurro”—had increased 773.820% and 715%, respectively. In Spain, streams of “Resistiré” increased by 435%. “Abbracciame” (“Embrace Me”), by Andrea Sannino and first released in 2015, has since earned the “gold” certification by Federazione Industria Musicale Italiana in August 2020, with 35,000 copies sold; at that time, it had received over 41 million views on YouTube and 7 million streams on Spotify. Undoubtedly, balcony-singing has had an immense impact on musical culture during the COVID lockdowns and will remain, for many, one of the musical practices indelibly associated with that period.

During lockdowns, participants in musical activities such as flash mobs or online ensembles as well as their spectators have frequently reported the alleviation of stress and the feeling of connectedness as a result of their musical engagement. Erica Marino was a participant in one of the earliest balcony flash mobs in Benevento, the video of which went viral in early March. In an interview with Fanpage.it about her participation, Marino said, “We were all down and a bit worried about the situation we were living through, and this was a spontaneous gesture of release, of happiness, and to raise our spirits for a moment. And it did us good, I must say. It did good for the neighborhood, because they looked out, they greeted and thanked us, they finally opened their windows… It was a gesture of release for me, to take the drum and go out—a call to life, to joy, and to music, which is my greatest passion. I dance folk dances of southern Italy, and these days, I am dancing at home, so this was a moment of release shared with the neighbors” (Cozzolino, 2020). When asked about the messages flooding in from all over Italy after the video of their music making became widely shared on social media, Marino reported that viewers expressed gratitude “because they saw in this video a message of hope and positivity, because going onto Facebook and other social networks is devastating, because there is only news of people with panic attacks, fake news… and because there is only terrifying news. Playing took me out of reality for a moment” (Cozzolino, 2020). In short, participating in (and watching) these musical performances can fulfill the CDC’s (and Renaissance doctors’) recommendations for setting aside fear and pursuing recreational activities with friends and loved ones.

Balcony-singing during lockdown, in fact, has deep roots. In 1576, when the Milanese plague grew more deadly through October and general quarantine was imposed, public processions came to an end—on the streets, at least. Borromeo relocated the ritual inside private homes, decreeing that church bells across the city were to be rung seven times a day, every 2 h, and “while the bell is rung, litanies or supplications will be sung or recited at the direction of the Bishop. This will be performed in such a way that one group sings from the windows or the doors of their homes, and then another group sings and responds in turn” (Borromeo, 1595, p. 55–66). Borromeo’s spiritual “deputies” were placed on street corners to initiate and moderate this devotion.

Like many of us who were impressed by our balcony singers, a number of chroniclers were also moved by the Milanese ritual. Paolo Bisciola wistfully documented:

[W]hen the plague began to grow, this practice [of singing the litanies in public] was interrupted, so as not to allow the congregations to provide it more fuel. The orations did not stop, however, because each person stood in his house at the window or door and made them from there […] Just think, in walking around Milan, one heard nothing but song, veneration of God, and supplication to the saints, such that one almost wished for these tribulations to last longer (Bisciola, 1630, 3v–4r).

Highlighting the idea of unity and communal solidarity, Borromeo’s biographer Giovanni Pietro Giussano recalled, “It was a sight to see, when all the inhabitants of this populous city… united to praise God at one and the same time, sending up together a harmonious voice of supplication for deliverance from their distress.” He then compared the Milanese to nuns and monks “serving God in the enclosure of their cells,” and the entire city to “the heavenly Jerusalem filled with the praises of the angelic hosts” (Giussano, 1884, p. 419–20).

This ritual of singing separately together earned the approval of doctors as well. In his treatise on the plague, composed a decade after the Milanese outbreak, Francesco Alessandri warned that “any unnecessary gatherings of people, [in] schools, universities, markets, dances, banquets, and churches should be prohibited.” Furthermore, he declared processions to be unsafe, “even though it is always good to be devout, and especially in such a time.” As a compromise, he suggested, “it is enough that, at the seventh peal of the bell every day, people of both sexes, of every age kneel at home and say the psalms of David, or indeed that they pray to the lord, responding to the litany from street to street” (Alessandri, 1586, 30v).

If we return to the instances of balcony singing today, we can see that while the religious aspect of these ad hoc musical sessions has been attenuated, the civic orientation has not. Some recorded instances show a clear performer vs. audience relationship, but more frequently, communal participation is involved. Certain choices of music have facilitated such engagement. Recent pop hits, certainly, but also dance music and songs with well-known choreography such as the “Macarena” allow participants to respond, even in a non-strictly musical way. Music such as the tammurriata, which traditionally calls for drumming, allows for the use of makeshift percussion instruments (one Italian balcony performer was recorded in a New Yorker video exhorting her neighbors, “All you need is two wooden spoons and a pan, and you can play whatever”; Taladrid, 2020). In that vein, some communities use bell-ringing for a similar purpose.

Many communities have also used songs with local significance, such as national anthems and music by local artists. For example, Montrealers sang songs by Leonard Cohen, whom The New York Times had dubbed “the New Secular Saint of Montreal” (Bilefsky, 2018). Folk songs and “unofficial anthems” have also been useful: “Vesuvio” carries obvious geographical significance for the Beneventans, and the Sienese sang “Il Canto della verbena,” a song that references the verbena leaves that grow in the city’s historic center and is sung regularly at other civic festivities, including the twice-annual *Palio* horseraces (Paradis, 2019). On 13 March 2020, Milanese trumpeter Raffaele Kohler performed “O mia bela Madunina” on his instrument at his window. Like “Il Canto della verbena,” “O mia bela Madunina” is a local anthem. The chorus of the song addresses the statue of Mary atop the Duomo: “O, my beautiful Madunina, you who shines from afar, all golden and small, you rule over Milan. So come without fear, we will extend our hand to you. The entire world is a village, indeed, but Milan is a great Milan.” In an interview with the local paper *Il Girono*, Kohler explained that he chose “O mia bela Madunina” to “give strength to our city, so that the melody that is so dear to us can improve our morale, helping us to overcome this critical moment” (Vazzana, 2020). Shouts of “forza Milano” heard after Kohler’s performance demonstrate the desired effect.

With technology, the “call-and-response” of such performances becomes geographically boundless. Inspired by Kohler’s performance (and by a video of Archbishop Mario Delpini on the roof of the cathedral, facing the Marian statue and reciting a litany-like prayer that uses “O mia bela Madunina che te dominet Milan” as a refrain), the Italian band Blues4People and members of the American Blues Brothers collaboratively produced a contrafact of the song a few weeks later as an expression of trans-Atlantic solidarity. In the preamble to the video, called “The Blues Bunker Session,” Lou Marini, a member of the original Blues Brothers says, “We are thinking about you guys in Italy. We are starting to go through the same thing in New York, so we wanted to send a little musical message to you.” The contrafact verses contain some tropes common in plague narratives (“A virus has now arrived from Asia and slowly plagued all Italy”—something also said of the Black Death), some advice that Renaissance doctors and the CDC would approve (“Stay home, play cards, and pray”; “We touch elbows, don’t shake hands anymore”), and interjections of encouragement. The very performance of the Milanese tune in an American blues idiom aptly symbolizes participant Carlo “Jake” Fumagalli’s description of music as a social “glue” (Vites, 2020). The Milanese sang from “street to street” in the sixteenth century. Today, we sing from continent to continent.

Balcony flash mobs may also provide participants—in addition to social solidarity and the therapeutic benefits of music—a way of regulating their time. There have been many anecdotal reports of people feeling that time is “broken” under lockdown. As Pardes (2020) described the phenomenon: “The virus has created its own clock, and in coronatime, there is less demarcation between a day and a week, a weekday and a weekend, the morning and night, the present and the recent past…[T]hese distortions of time feel strangely universal.” Research by psychologists into “coronatime” substantiate Pardes’ impression. In a study of the perceived passage of time during the UK lockdown, Ogden (2020) reported that—while not quite universal—80% of 604 survey respondents experienced distortions in their sense of time, with a nearly even split between those who felt time moving quicker and those who felt it moving slower. The age of the participants, satisfaction with levels of social interaction, and stress and task load were predictive of their experiences. Time passed more slowly for those who were over the age of 60, felt a greater level of stress, and had greater dissatisfaction with the amount of social interaction. Time passed more quickly for those who were younger, more socially satisfied, and had increased task loads. A French research team studying the same phenomenon found that boredom and sadness—not stress—correlated more reliably with the experience of a slowdown (Droit-Volet et al., 2020). Regardless of the cause of coronatime, musicians have reported looking forward to group music making as a way of regulating their schedules. One Italian participant described flash mobs as “a way to feel closer, a daily appointment to be social” (Taladrid, 2020). Canadian author Saleema Nawaz wrote in the *Globe and Mail*, “Like many of us, the entirety of my former schedule—meals, sleep, work—has been upended. I can’t seem to make one day resemble the next. Now choir practice is the only hour of my life that endures from before the pandemic. Thirty-odd familiar faces on my screen remind me I’m not alone” (Nawaz, 2020).

Early modern Europeans under quarantine may have experienced similar distortions of time. The English poet Abraham Holland, who succumbed to the plague that was the subject of his posthumous epic “A Description of the Great, Fearful, and Prodigious Plague, 1625,” described in that poem the confusion of the hours:

*Through the nak’d town, of death there was such plenty*,

One Bell at once was fain to ring for twenty.

*No Clocks were heard to strike upon their Bells*,

Cause nothing rung but death-lamenting Knells.

*Strange that the Hours should fail to tell the Day*,

When Time to thousands ran so fast away.

*Time was confus’d and kept at such a plight*,

The Day to thousands now was made a Night. (Cited in Totaro, 2016, p. 169)

Here, Holland uses the conceit of bells—the chimes of the clock are overtaken by death knells—to conflate daily time with the timespan of life and links the upheaval of mass casualties to the experience of distorted hours. Projecting our experiences to the past, it is possible to imagine how the Milanese regulated their time by coming to their windows to sing seven times a day, every 2 h, at the summon of a church bell.

## Conclusion

Even when we were in the state that Daston called “ground-zero empiricism,” we were not bereft of knowledge and experience. The fundamental tenets of science—in medicine, virology, laboratory methods—remained guiding principles. What our historical comparison shows is that neither were we bereft of time-honored tools, such as music, to help maintain our individual and collective wellbeing. Some of the continuities in musical activities across pandemics, even if the medical rationale or cultural imperatives and technologies supporting them have changed, can reveal what we have repeatedly found useful in music in disastrous times. In many of the activities cited, music fulfills the functions of mood regulation and social cohesion, often times together; Bocaccio’s bubblers and the Milanese inmates tempered their affects with social musical activities, just as balcony singers today cast off worries with their neighbors in song. Processions and litanies can link together communities whether through in-person synchronous actions (as described by Keller et al., 2014) or in spirit by recalling common struggles and common devotional histories, while playlists can help their creators connect with friends and form new virtual tribes.

It is perhaps not surprising that the mood maintenance and social cohesion functions of music emerge so clearly during epidemics past and present, given the sustained emotional turmoil and social isolation that result from widespread outbreaks of contagious diseases. Further research into music making under such extraordinary conditions may tell us more about the interrelationship between those two functions, as well as the changes in the general tactics and their relative successfulness for achieving them, on account of the necessary limitations in the range and modalities (in-person vs. virtual) of activities available. Furthermore, because global catastrophes are thankfully rare, COVID-19 provides a special opportunity to undertake cross-cultural comparisons of musical communities across the world that face the same medical threat and undergo similar public-health restrictions.

While historical evidence readily shows the use of music for mood maintenance and social cohesion, the use of music for achieving self-awareness in the Renaissance is less apparent. This lacuna of information arises both from the kinds of historical sources available and the manner in which early-modern Europeans described (or not) their subjective experiences of music, which is ultimately tied to a different notion of selfhood that is not easily reconcilable with the ways that selfhood is expressed today (see Greenblatt’s, 1980 work on Renaissance self-fashioning, for example). The nature of musical introspection under the conditions of COVID may, therefore, be a more contextually contingent avenue of study. Although much remains to be learned about our musical experiences under the COVID lockdowns, trans-historical investigations, yielding both continuities and discontinuities, can be a lens to sharpen our focus on the benefits we can derive from music for regulating the health of our biological and social bodies amid medical catastrophes.

## Author Contributions

The author confirms being the sole contributor of this work and has approved it for publication.

## Conflict of Interest

The author declares that the research was conducted in the absence of any commercial or financial relationships that could be construed as a potential conflict of interest.
